# Understanding Long-Term Outcomes Following Sepsis: Implications and Challenges

**DOI:** 10.1007/s11908-016-0544-7

**Published:** 2016-10-06

**Authors:** Manu Shankar-Hari, Gordon D. Rubenfeld

**Affiliations:** 1 Critical Care Medicine, Guy’s and St Thomas’ NHS Foundation Trust, 1st Floor, East Wing, St Thomas’ Hospital, London, SE17EH UK; 2 Division of Asthma, Allergy and Lung Biology, Kings College London, London, SE1 9RT UK; 3 Interdepartmental Division of Critical Care Medicine, Sunnybrook Health Sciences Centre, 2075 Bayview Avenue, D5 03, Toronto, ON M4N 3M5 Canada

**Keywords:** Sepsis, Epidemiology, Causality, Long-term outcomes, Morbidity, Mortality, Survivors

## Abstract

Sepsis is life-threating organ dysfunction due to infection. Incidence of sepsis is increasing and the short-term mortality is improving, generating more sepsis survivors. These sepsis survivors suffer from additional morbidities such as higher risk of readmissions, cardiovascular disease, cognitive impairment and of death, for years following index sepsis episode. In the first year following index sepsis episode, approximately 60 % of sepsis survivors have at least one rehospitalisation episode, which is most often due to infection and one in six sepsis survivors die. Sepsis survivors also have a higher risk of cognitive impairment and cardiovascular disease contributing to the reduced life expectancy seen in this population, when assessed with life table comparisons. For optimal design of interventional trials to reduce these bad outcomes in sepsis survivors, in-depth understanding of major risk factors for these morbid events, their modifiability and a causal relationship to the pathobiology of sepsis is essential. This review highlights the recent advances, clinical and methodological challenges in our understanding of these morbid events in sepsis survivors.

## Introduction

Sepsis has been redefined recently as life-threatening organ dysfunction caused by dysregulated host responses to infection and septic shock as a subset of sepsis in which particularly profound circulatory, cellular and metabolic abnormalities are associated with a greater risk of mortality than with sepsis alone [1, 2].

Globally, sepsis is common, with an estimated population incidence of 270 (95 % CI 176–412) cases per-100,000 person-years and acute mortality of 26.0 %[3]. A number of reasons suggest even this underestimates the magnitude of sepsis associated mortality and morbidity. First, as the authors’ of this paper [3] highlight, the incidence data is primarily critical care based, with limited data from low- and middle-income-countries. Second, at the bedside, sepsis cases represent either a new organ dysfunction or worsening of chronic organ dysfunction such as those seen in comorbid conditions [1], in the context of suspected or proven infection. The literature on prevalence of organ dysfunction outside the critical care environment is limited and when estimated appears frequently [4]. Alongside this underestimated incidence globally, the short-term mortality from sepsis is improving [5, 6]. This epidemiology pattern generates approximately 14 million sepsis survivors globally [3], increasing yearly, with ongoing health care needs [7].

In this background, after highlighting the conceptual approach and methodological challenges, this review focuses on the additional long-term risk of death, readmissions, cardiovascular disease, cognitive impairment and quality of life (QOL) alterations in sepsis survivors, followed by a brief overview of biological mechanisms contributing to these outcomes.

### Conceptual Approach

**Fig. 1:**
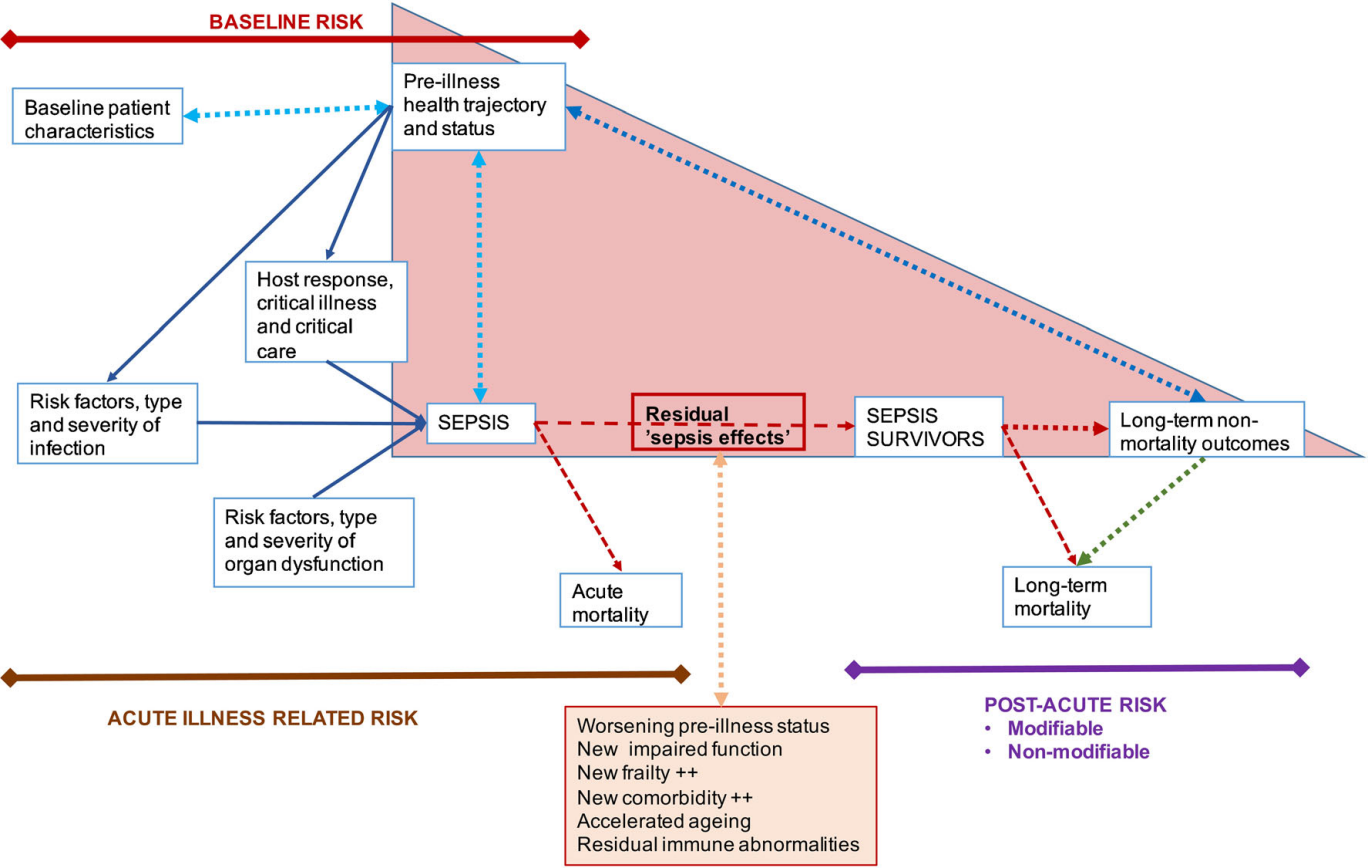
Conceptual framework for multiple interacting factors influencing sepsis associated long-term outcomes. The *red triangle* highlights the vicious cycle between pre-illness morbidity—sepsis—long-term outcomes pathway. The *arrows* represent direction of relationship between factors. Sepsis occurs in healthy and in subjects with co-morbidities, acutely altering pre-event health state. Thus, complex interactions between pre-sepsis health state and additional morbidity and frailty related to sepsis illness influence post-sepsis health state in sepsis-survivors

Sepsis and the outcomes following sepsis are best conceptualized as consequences of a complex interplay between baseline characteristics including pre-illness health status, risk factors for infection, dysregulated immune responses and those for developing acute organ dysfunction, health care setting, treatments provided and the response to treatments. The risk factors for infection include extremes of age, male sex, comorbidities, race, genetics, prior sepsis, surgery, any hospitalisation and frailty [8–16]. There is no accepted definition for dysregulated immune responses in sepsis [17], the risk factors for infection highlighted and potentially genetic variations [18] determine these immune responses. The mechanisms of organ dysfunction in sepsis are still debated [19, 20]; the risk factors for infection and immune responses are considered risk factors for organ dysfunction. In addition to short-term outcomes, all these characteristics also influence the long-term outcomes in sepsis survivors [21–24, 25••]. One of the most important lessons from the last decade of studying long-term outcomes of sepsis and other critical illnesses is that poor functional status is a risk factor for becoming critically ill as well as a frequent consequence. Similarly, many comorbidities, age and chronic diseases are risk factors both for sepsis and for impaired quality of life. Therefore, it is important in reviewing the literature to distinguish studies that have tried to separate out the potentially causal effects of sepsis from those that simply describe morbidity and mortality events [25••]. Understanding this fundamental concept helps to identify risk factors including those that are changeable, quantify this modifiable risk and target specific interventions at specific time-points in survivor trajectory to improve health in sepsis survivors (Fig. 1).

### Challenges

There are a number of challenges to studying the potentially causal post-acute residual effects of sepsis. First, when studying risk factors and assessing modifiable risk, the acute effects of sepsis have to be separated by specifically looking for independent associations in the sepsis survivors population as opposed to the admission cohort with sepsis. Second, dose-response effect is important for biological plausibility arguments. An increase in sepsis severity may worsen post-acute sequelae, however, it will also increase the probability of short-term mortality. Therefore, the competing risk of death may hide the longer-term non-mortality effects of sepsis. Third, studies evaluating the long-term outcomes following sepsis often do not account for pre-illness trajectory. When this is accounted for, the independent effects of sepsis on long-term outcomes generate different results [9]. Fourth, studies without control arms will not provide an estimate of sepsis attributable risk [25••, 26••] and therefore are of little value in planning interventions. Fifth, if the long-term outcome studied cannot be defined for all individuals in a cohort either due to death or loss to follow-up and this missing data is differential, bias due to truncation-by-death occurs [27]. Typically, this occurs when evaluating long-term quality of life in survivors using follow up data from clinical trials and observational studies, when there is missing data from those sepsis survivors who die prior to obtaining a quality of life estimate. In the scenario where trial data is used, the probability of death in itself may be altered by the intervention and the baseline balance of randomisation is lost when assessing survivors. In observational studies, those survivors with missing data may be systematically different to those who have follow up data.

### Methodological Approaches to Overcome Challenges

There are two ideal study designs to measure the causal effects of sepsis on long-term outcomes: a yet to be done large cohort study starting with a healthy population well before index sepsis episode to understand pre-illness trajectory, then these patients are followed through after sepsis with detailed measures of health status, processes of care, and biologic measures or an impossible controlled trial that randomly induces sepsis. The available observational studies use alternative methods to address this question with attendant limitations. To ascertain the pre-illness trajectory, a health care contact look back could be done and a look back period between one and 5 years is considered appropriate [28]. To account for bias secondary to lack of randomisation, there are numerous methods, the most well recognised being propensity score based matching. This approach compares the outcomes of patients who get septic with controls who were equally likely to get septic but did not [29]. The principal limitation being unobserved confounders may vary even after adjustment and matching. When studying long-term effects of sepsis, there are time-varying confounders and mediators such as age, comorbid conditions, chronic medications, ageing, frailty. Marginal structural models (MSMs) provide population-averaged causal effects, which go beyond the statistically independent associations generated by regression models and post estimation statistics [30]. Studies without control populations (patients without sepsis as exposure), sepsis attributable risk is imperceptible and is prone to inference bias if the control population chosen is not similar to sepsis cohort in all aspects except for the exposure (sepsis). This important concept is highlighted using cardiovascular events in sepsis survivors, and it is this attributable risk is what we hope to reduce using interventional studies.

### Long-Term Outcomes

#### Mortality in Sepsis Survivors

Using fundamental principles of causality [31], we recently reported the limited strength of epidemiological evidence supporting the premise that sepsis causes an increase in all-cause mortality after hospital discharge (post-acute mortality) [25••]. Although the 1-year post-acute mortality was 16.1 % (95%CI =14.1–18.1 %; N = 43 studies), there was evidence of statistical and clinical heterogeneity, bias and residual confounding. Post-acute mortality was associated with age, male, co-morbidities, and deterioration in health prior to sepsis. Since publication of our systematic review, there has been two further publications [32•, 33•]. The well-conducted study by Prescott et al. focussed on patients older than 65 years and used double robust analysis [34] with a regression model for mortality and propensity score to account for covariates to show that sepsis is an independent risk factor for post-acute mortality [32•]. Using propensity score matching, Ou et al. also showed an increase in all cause mortality in sepsis survivors [33•]. Sepsis associated increased risk of death extends for up to ten years following index hospitalisation [25••, 32•, 33•, 35].

The magnitude of sepsis-post-acute mortality association varied dependent on the control population chosen for comparison. The additional hazard associated with sepsis was greatest when compared with general populations [25••]. The magnitude of additional risk of death in sepsis survivors decreased when the selected control population had more severe non-sepsis illnesses. The major implication being that the literature currently over estimates the risk of post-acute death caused by sepsis and therefore preventable fraction is likely to be lower, which needs to be accounted for during sample size estimation in future RCTs designed to reduce late mortality in sepsis survivors.

#### Rehospitalisation in Sepsis Survivors

Compared to non-sepsis admissions, sepsis survivors had a greater risk of rehospitalisation [10, 36–38], and rehospitalisation increases the risk of death. The average 30-day rehospitalisation rates in sepsis survivors are between 19.9 and 32 % [7, 36–43]. The cumulative rehospitalisation rates increases with follow-up time with the 90-day and 1-year rehospitalisation rates being 40 % [7, 36] and 63.0 % [7], respectively, implying persistent risk in sepsis survivors. Pneumonia survivors have lower 30-day rehospitalisation rates (16.5 % [44] and 18.2 % [45••]), implying potentially a dose-response effect.

The additional readmissions risk was significantly more common in sepsis survivors with matched hospitalisations with acute medical conditions comparisons and a proportion of these readmissions are potentially preventable [36]. In a retrospective cohort study, except for parenteral nutrition, the other risk factors for readmissions identified in univariate analyses such as transfusion, duration of antibiotics, disappeared after regression analyses to account for confounding [38]. In contrast, 30-day rehospitalisation after sepsis has been shown to be independently associated included age, malignancy diagnosis, hospitalizations in the year prior to sepsis admission and low haemoglobin concentrations at discharge [37].

The most common reason for rehospitalisation in sepsis survivors was infection [46] and infection-related rehospitalisation represented either unresolved/recurrent infection or new infections [38]. The risk factors and magnitude of infection-related readmissions differed based on definition used, implying clinical heterogeneity and confounding by indication in studies. Infection-related rehospitalisation was independently associated with index admission pathogens (multidrug resistant pathogens, *E. Coli* spp. and *Bacteroides* spp. infections), renal dysfunction and urinary tract source, with *E Coli* spp. and urinary tract being protective [43]. The most common site in infection-related rehospitalisation in sepsis survivors was pneumonia, whereas it was urosepsis in non-sepsis survivor rehospitalisation [46]. In this study, other independent risk factors for infection-related rehospitalisation were prolonged hospitalization, age and the presence of an indwelling catheter [46], implying admission case-mix is an important determinant for infected-related rehospitalisation in sepsis survivors.

As with post-acute mortality, further research is required to understand the true preventable fraction and modifiable risk factors for rehospitalisation, in particular infection-related rehospitalisation. Most studies censor death, which is a competing risk factor for readmissions. Independent associations generated by regression models represent strength of this relationship not a causal pathway, which is yet to be proven. The duration of additional risk of infection-related rehospitalisation and the underpinning mechanisms needs to be further characterised.

#### Cognitive Impairment in Sepsis Survivors

Hospitalization, regardless of aetiology, is associated with cognitive decline [47, 48]. The relationship between sepsis and cognitive decline is likely to be complex and bidirectional as pre-illness cognitive decline is a risk factor for pneumonia and sepsis [9, 49, 50••] and sepsis is also an independent risk factor for cognitive decline (OR 95 % CI 3.3 (1.5–7.3)) [49]. Thus, there is a threefold increase in risk of cognitive decline in sepsis survivors compared to control populations, but the rate of new cognitive decline in those sepsis survivors is similar to that seen with pre-illness deterioration [49]. Similarly, pneumonia is associated with cognitive decline and increases the hazard for dementia (OR 95 % CI 2.2 (1.6–3.6)) [50••]. This hazard ratio does not vary with sepsis severity, implying no dose response effect [50••]. Depression, which is common in sepsis survivors, is also a risk factor for pneumonia [51] and sepsis [52] and is significantly associated with post sepsis functional impairment [52].

Delirium is common during critical illness, prolongs hospitalisation and has minimal attributable mortality [53•]. Acute delirium is also associated with cognitive impairment including dementia and depression during longer-term follow-up of critical illness survivors [54, 55] and in hospitalised patients [56]. Sepsis is associated with delirium [57] and pre-sepsis episode cognitive decline. Thus, the post-sepsis cognitive decline could either have a causal relationship with sepsis or could be unrelated to sepsis but acting via the delirium pathway. A related concept that is difficult to test with new-onset cognitive decline or dementia is the extent of cognitive reserve, whether and how this reserve is modified by sepsis. This is important as incident cognitive decline is related to cognitive reserve and depends on follow-up duration [58].

#### Cardiovascular Complications in Sepsis Survivors

In the last 3 years, numerous studies have reported the long-term risk of cardiovascular events in sepsis survivors. Corrales-Medina et al. followed two community-based cohorts (>65 years of age and 45–64 years of age) and assessed the relationship between index pneumonia episode and subsequent cardiovascular disease (CVD) events over a 10-year period [59]. In this study, CVD events were myocardial infarction, stroke and fatal coronary heart disease. The older cohort had greater CVD events (34.9 % compared to 16.5 %). In both cohorts, sepsis was an independent risk factor for CVD events and the risk was greatest in the year following index pneumonia episode [59]. Of note, there were residual imbalances in covariates (e.g. hypertension, diabetes mellitus, smoking), despite incidence density sampling used to match cases to controls in this study [59]. Using propensity score based matching; Yende et al. assessed the independent risk of cardiovascular events after sepsis as an explanation for long term increased mortality risk in sepsis survivors [26••]. The primary outcome was the 1-year incidence rate of hospitalised cardiovascular events in sepsis survivors. Sepsis survivors had statistically significant higher rate of cardiovascular events when compared to propensity matched critically ill, hospitalised infected controls, and hospitalised non-infected controls. However, the magnitude of risk attributable to sepsis was minimal when cardiovascular event risks of acute care and baseline characteristics were accounted for [26••]. Jafarzadeh et al. assessed the cumulative 5-year risk of cardiovascular events in sepsis and used MSMs to generate a causal inference of additional risk. The odds ratio for sepsis associated any CVD was 2.39 (1.88–3.03), and there was a dose response with increase in severity (Bacteremia = 1.52 (1.21–1.90); Sepsis = 3.60 (2.59–5.00); and Septic shock = 4.55 (3.58–5.78)). In a community acquired bacteremia cohort from Denmark with matched general population controls and hospital controls, Dalager-Pedersen et al. show that the risk of myocardial infarction is greatest in the first 30-days following bacteremia. This independent association disappears by 6 months following index bacteremia [60]. Similar duration of additional risk for CVD events has been reported in other cohorts [33•, 61].

#### Quality of Life in Sepsis Survivors

Winters et al. performed a systematic review and identified 12 studies that used different validated tools to assess the QOL in sepsis survivors, the most common being Short Form 36 (SF-36) [62]. Overall, sepsis survivors have impaired QOL compared to population norms [62, 63] and this impaired QOL lasts as long as 5-years after index admission [64]. To account for pre-illness status and truncation-by-death issues highlighted earlier, Yende et al. used patients enrolled in two randomised controlled trials and who lived independently prior to the index sepsis episode to show impaired QOL in sepsis survivors [65]. However, the SF-36 scores in sepsis survivors were similar to patients with comorbid conditions such as chronic obstructive pulmonary disease, hypertension and congestive heart failure [66]. Sepsis survivors also have similar QOL to non-sepsis critically ill survivors [64, 66–68]. Prehospital QOL appears to be an important determinant of QOL after discharge following hospitalisation and the magnitude of additional risk due to critical illness is small compared to all cause hospitalisations [69]. These observations challenge a causal inference of sepsis effect on QOL.

#### Biological Explanations for Long-Term Effects in Sepsis Survivors

Despite the robustness of statistical methods and independent associations with some of the long-term outcomes following sepsis, the epidemiological causality and biological mechanisms are yet to be proven. The cause of death in sepsis survivors is seldom reported, and the mechanisms leading to accelerated death in sepsis survivors, if this observation is causal, are uncertain. Sepsis often occurs in patients with comorbid conditions [13, 70], frail and elderly [8]. Many of the long-term outcomes reported in sepsis survivors are also common in elderly and frail populations, implying accelerated ageing could be a potential mechanism. Aging is a multisystem process characterised by cell damage, responses to these cell damage events and the ensuing phenotype. The cell damage is considered secondary to genomic instability, telomere attrition and epigenetic alterations [71], which is a nascent literature in sepsis [72–74]. The cell damage events seen in aging results in abnormalities of nutrient sensing, mitochondrial function and cellular senesce [71], some of which are also seen in sepsis. Ageing results in impaired lymphopoiesis with relative preservation of myelopoiesis, with reduction in stem cells. The T cell memory pool is increased, with skewed expansion of CD8 T cells and CD4 differentiation into Th17 population with reduced B cell diversity. The lymphocytes from sepsis patients share a number of these features [75], implying accelerated aging of the immune system in sepsis.

In well-characterised caecal ligation and puncture murine models of sepsis, sepsis results in sustained central nervous system inflammation after recovery, which could explain the long-term cognitive impairments reported [76]. In similar murine models, elevated serum levels of high mobility group box 1 (HMGB1) was associated with cognitive dysfunction [77] and is hypothesised to act via the up regulated receptor for advanced glycation end products (RAGE) [78]. Thus, the hypothesis that neuronal inflammation in sepsis survivors explains cognitive impairment should be studied. Kaynar et al. did a RCT using CLP sepsis models in murines predisposed to atherosclerosis (ApoE-deficient) and with wild-type mice [79]. The authors showed that sepsis resulted in persistent inflammation that predisposes to accelerated atherosclerosis [79], which provides an explanation for CVS risk in sepsis survivors in addition to baseline demographic and other risk factors. In the future, understanding of mechanisms underpinning this accelerated atherosclerosis may identify potential intervention targets.

Readmissions due to infections in sepsis survivors could be either due to impaired immune system functions and/or imbalances in the gastrointestinal microbial flora (dysbiosis) in sepsis. The acute immune responses in sepsis characterised using pan leukocyte transcriptome profiles show activation of pro-inflammatory and anti-inflammatory pathways in the innate and adaptive immune systems and leaves residual immunosuppression in sepsis survivors [17, 80–82]. The mechanisms for post-acute immunosuppression in sepsis survivors are not well understood. To date, one pilot study involving just *eight* patients highlight abnormalities in T cells and impaired cytokines responses to extrinsic stimuli, long-after index sepsis episode [83]. Aside from the small sample size, the variable time interval from index sepsis episode to immune assessments (between 9 and 60 months) and high risk of bias preclude meaningful inferences. Similar changes were also observed in splenocytes and lung parenchyma in a landmark paper studying deaths from protracted sepsis [84]. A healthy microbiome consists of obligate anaerobes (e.g. Bacteroidetes, Firmicutes), and facultative anaerobes (e.g. Proteobacteria) with diverse with metabolic functions that maintain check on pathological bacterial density [85–87]. Acute illness, hospitalisation and antibiotics alter this balance towards a simpler and potentially pathogenic gut bacterial flora alongside increase in risk for Clostridium Difficile infections [87–89]. In a study that did not provide any microbiological evidence but presumed dysbiosis based on the microbiome literature, suggested dysbiosis as a risk factor for infection related rehospitalisation in sepsis survivors [10]. Thus, it is valid to hypothesise that both immunosuppression and dysbiosis in sepsis survivors potentially contribute to infection related rehospitalisation. Further studies are required to confirm these hypotheses.

## Conclusions

Sepsis survivors have a different health trajectory prior to and following their acute illness, but the potentially causal role of sepsis on the observed long-term impairment and survival remains unclear. For some clinicians and health care administrators, the question of causality is irrelevant. It is a fact that survivors of sepsis, as well as many other acute illnesses requiring hospitalization, are profoundly functionally impaired and their health care needs as well as their caregivers’ needs must be addressed. However, for investigators designing interventions to prevent or treat these long-term sequelae, a fundamental understanding of the clinical and biological mechanisms causing these long-term morbid events in sepsis survivors is required.
